# Methods for Social Media Monitoring Related to Vaccination: Systematic Scoping Review

**DOI:** 10.2196/17149

**Published:** 2021-02-08

**Authors:** Emilie Karafillakis, Sam Martin, Clarissa Simas, Kate Olsson, Judit Takacs, Sara Dada, Heidi Jane Larson

**Affiliations:** 1 London School of Hygiene & Tropical Medicine Vaccine Confidence Project London United Kingdom; 2 European Centre for Disease Prevention and Control Stockhom Sweden; 3 Centre for Social Sciences Hungarian Academy of Sciences Budapest Hungary; 4 Institute for Health Metrics and Evaluation University of Washington Seattle, WA United States

**Keywords:** vaccination, antivaccination movement, vaccination refusal, social media, internet, research design, review, media monitoring, social listening, infodemiology, infoveillance

## Abstract

**Background:**

Social media has changed the communication landscape, exposing individuals to an ever-growing amount of information while also allowing them to create and share content. Although vaccine skepticism is not new, social media has amplified public concerns and facilitated their spread globally. Multiple studies have been conducted to monitor vaccination discussions on social media. However, there is currently insufficient evidence on the best methods to perform social media monitoring.

**Objective:**

The aim of this study was to identify the methods most commonly used for monitoring vaccination-related topics on different social media platforms, along with their effectiveness and limitations.

**Methods:**

A systematic scoping review was conducted by applying a comprehensive search strategy to multiple databases in December 2018. The articles’ titles, abstracts, and full texts were screened by two reviewers using inclusion and exclusion criteria. After data extraction, a descriptive analysis was performed to summarize the methods used to monitor and analyze social media, including data extraction tools; ethical considerations; search strategies; periods monitored; geolocalization of content; and sentiments, content, and reach analyses.

**Results:**

This review identified 86 articles on social media monitoring of vaccination, most of which were published after 2015. Although 35 out of the 86 studies used manual browser search tools to collect data from social media, this was time-consuming and only allowed for the analysis of small samples compared to social media application program interfaces or automated monitoring tools. Although simple search strategies were considered less precise, only 10 out of the 86 studies used comprehensive lists of keywords (eg, with hashtags or words related to specific events or concerns). Partly due to privacy settings, geolocalization of data was extremely difficult to obtain, limiting the possibility of performing country-specific analyses. Finally, 20 out of the 86 studies performed trend or content analyses, whereas most of the studies (70%, 60/86) analyzed sentiments toward vaccination. Automated sentiment analyses, performed using leverage, supervised machine learning, or automated software, were fast and provided strong and accurate results. Most studies focused on negative (n=33) and positive (n=31) sentiments toward vaccination, and may have failed to capture the nuances and complexity of emotions around vaccination. Finally, 49 out of the 86 studies determined the reach of social media posts by looking at numbers of followers and engagement (eg, retweets, shares, likes).

**Conclusions:**

Social media monitoring still constitutes a new means to research and understand public sentiments around vaccination. A wide range of methods are currently used by researchers. Future research should focus on evaluating these methods to offer more evidence and support the development of social media monitoring as a valuable research design.

## Introduction

Although public questioning of vaccination is as old as vaccination itself [1], continuous advancements in the global communication landscape, associated with the rise of social media as an interactive health information ecosystem, have contributed to the unmediated spread of vaccine hesitancy [2]. This new boundless information ecosystem has shaped the nature of conversations about vaccination, with evidence showing that social media can facilitate the quick diffusion of negative sentiments and misinformation about vaccination [2-6]. Furthermore, individuals have been found to more commonly engage with negative information around vaccination than positive content [7-10]. In this context, public trust in information provided by authorities and experts can decrease [11-13], influencing vaccine decisions [14]. Recent evidence has shown that social media users tend to cluster and create so-called “echo chambers” based on their views toward vaccination [15]; however, Leask et al [16] highlight that “a patient’s trust in the source of information may be more important than what is in the information,” stressing the importance of reaching individuals, across all clusters, through trustworthy sources.

Social media monitoring (infoveillance) provides opportunities to listen, in real time, to online narratives about vaccines, and to detect changes in sentiments and confidence early [17]. Information gathered from social media monitoring is crucial to inform the development of targeted and audience-focused communication strategies to maintain or rebuild trust in vaccination [17,18]. However, as social media monitoring can be resource- and time-intensive, and can raise issues of confidentiality, transparency, and privacy [17,19,20], evidence of public health communities investing in such listening mechanisms remains sparse.

The aim of this scoping review was to systematically summarize the methodologies that have been used to monitor and analyze social media on vaccination using an innovative three-step model. The findings presented in this paper come from a broader European Centre for Disease Prevention and Control (ECDC) technical report [21]. The aim of the ECDC report was to provide guidance for public health agencies to monitor and engage with social media, whereas this paper primarily focuses on the academic implications of social media monitoring. The specific objectives of this scoping review were to (1) identify the methods most commonly used for monitoring different social media platforms; and (2) identify the extent to which methods have been evaluated, along with their effectiveness and limitations.

## Methods

### Design

Systematic scoping reviews are used to map international literature with the aim of clarifying “working definitions and conceptual boundaries of a topic or field” [22] as well as identifying how research is conducted [22-24]. Systematic scoping reviews focus on scoping larger, more complex, and heterogeneous topics than systematic literature reviews. A systematic scoping review approach was therefore adapted to fulfill the goal of summarizing study methodologies used to monitor social media content around vaccination. The methodology for this scoping review was based on the work of Arksey et al [23] and Peters et al [24].

### Framing Social Media

Kaplan et al [25] define social media as “a group of internet-based applications that build on the ideological and technological foundations of Web 2.0, and that allow the creation and exchange of user generated content.” They further classify social media into blogs, collaborative projects (eg, Wikipedia), social networking sites (eg, Facebook), content communities (eg, YouTube), virtual social worlds (eg, Second Life), and virtual game worlds (eg, World of Warcraft) [25].

However, social media is not merely an information tool but also represents a continuously evolving social environment directly influenced by how individuals produce and share content, and interact with each other. For the purpose of this scoping review, we consider social media as not simply a means of communication but further a space within which individuals socialize and organize. This review therefore focuses on social networking sites and content communities, and excludes online platforms that do not have social interactions as their main purpose (eg, blogs or websites with a comments section).

### Search Strategy and Screening Process

The search strategy for the scoping review was developed by librarians at ECDC and researchers at the Vaccine Confidence Project (VCP), and was peer-reviewed to balance feasibility and comprehensiveness, including both social media and vaccination-related English keywords (see Multimedia Appendix 1). The search was conducted by one VCP researcher on the EMBASE database, and was adapted to search the PubMed, Scopus, MEDLINE, PsycINFO, PubPsych, Open Grey, and Web of Science databases in December 2018.

Identified articles were exported into Endnote, and duplicates were removed based on ECDC guidelines consisting of 6 rounds of deduplication looking for articles with similar author, year, and title; title, volume, and pages; author, volume, and pages; year, volume, issue, and pages; title; and author and year. The automated deduplication function in Endnote was not used, and articles were compared visually to ensure that only true duplicates were removed. Two VCP reviewers independently screened articles by title and abstract and by full text using a set of predefined inclusion and exclusion criteria. Disagreements were resolved by discussion.

Articles were included if they described studies performed to monitor or analyze data collected from social media around vaccination. The definition of social media described above was used as one of the inclusion criteria, limiting results to social networking sites and content communities. No restrictions were made with respect to location or language, as a team of official translators was available at the ECDC.

Articles were excluded if they were published before 2000 or if they were not about human vaccines. Articles that monitored online media (eg, news, websites) but did not collect any data from social media were excluded. The following article types were also excluded: conference abstracts, editorials, commentaries, and letters to the editor.

### Data Management and Analysis

Two VCP researchers extracted the following data from the included articles: country, aim, study population, period of monitoring, vaccine, social media, media monitoring methodologies (tool for data collection, keywords, exclusion criteria, geolocation), analysis (sentiment coding and analysis, reach, spread and interaction analyses, other types of analyses), results (number of posts), and evaluation and limitations.

**Figure 1:**
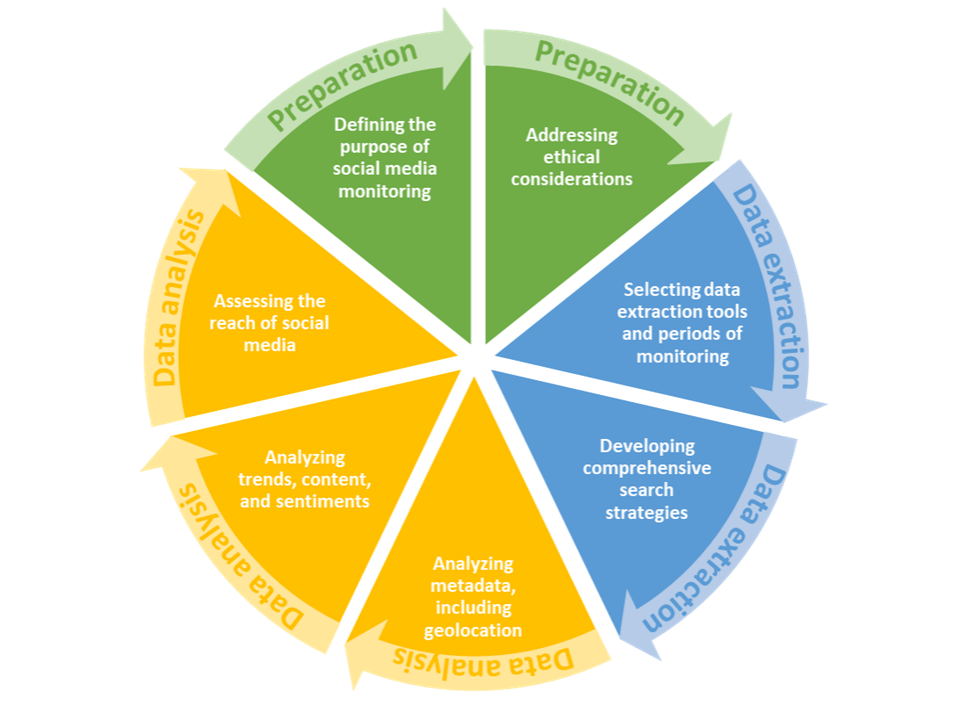
The three-step model of social media monitoring.

To facilitate the description of social media monitoring methods, a three-step model of social media monitoring was developed (Figure 1), including (1) preparation, (2) data extraction, and (3) data analysis steps. The preparation phase consists of defining the purpose of social media monitoring and addressing any ethical considerations. The data extraction phase includes selecting data extraction tools and periods of monitoring, developing comprehensive search strategies, and extracting the data. Finally, the data analysis stage includes geolocation, trends, content, sentiments, and reach analyses. The findings summarized in this paper are organized according to this three-step model.

Three researchers summarized, charted, and analyzed the data. A descriptive analysis was conducted for the types of data collection tools used to gather data from social media, the keywords and search strategies used, and the various analytical methods. These researchers reviewed and compared results in the data extraction sheet, listed and identified the frequency of different methods used for social media monitoring, and identified common themes. Two researchers met to discuss the findings and interpret them together with contextual information, identifying needs for further research.

## Results

### Included Studies

**Figure 2:**
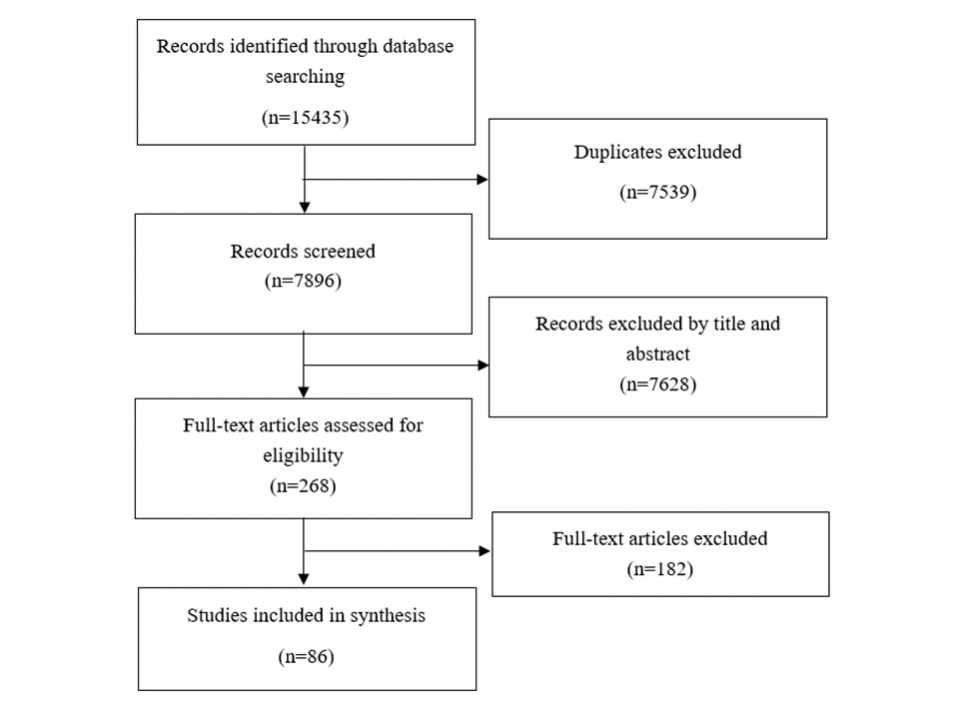
PRISMA (Preferred Reporting Items for Systematic Reviews and Meta-Analyses) flowchart.

The search strategy generated 15,435 articles, from which 7539 duplicates and 7628 irrelevant articles were excluded after screening by title and abstract (Figure 2). From the 268 articles screened by full text, 182 were excluded for the following reasons: not about social media (n=141); no data provided (n=19); conference abstracts, editorials, or letters to the editor (n=6); article not accessible (even after enquiring multiple libraries and contacting authors) (n=4); article containing data already published in another included article (n=1); and not on vaccination (n=1). At the end of the screening process, 86 articles in English, Spanish, and Italian were included for analysis. Articles in Spanish and Italian were analyzed by a researcher fluent in these two languages.

### Study Characteristics

The first study identified on social media monitoring around vaccination was published in 2006 [26], with an increasing number of studies published yearly since then. Most studies analyzed online discourse on Twitter (n=42) [8,27-67], YouTube (n=12) [68-79], Facebook (n=11) [80-90], and online forums (eg, babytree, mothering.com, mumsnet, KaksPlus; n=9) [26,91-98]. A diversification of social media platforms can be observed in recent years, with studies of platforms such as Pinterest (n=1) [99], Weibo (n=1) [100], Reddit (n=1) [101], or Yahoo! Answers (n=2) [102,103] all published after 2015. Seven studies monitored a mix of social media platforms [104-110]. More detailed study characteristics are provided in Multimedia Appendix 2.

### Social Media Monitoring Methods

A range of methods were used across the 86 included studies to monitor social media, most of which have not been evaluated in terms of their accuracy and reliability. In the following sections, these methods are described based on the three steps of social media monitoring proposed in this paper: preparation, data extraction, and data analysis.

### Preparation Phase

#### Defining the Purpose of Social Media Monitoring

The main objective of the majority of studies in this review (55/86, 64%) was to better understand how vaccination is portrayed on social media, whether through the analysis of online discourse or sentiments, or by looking at how information is produced, shared, and engaged with [8,26,27,30,33, 39,42-44,46-51,53-56,58-62,68-80,82-88,90-94,98,99,101,102,105,106].

Many studies (15/86, 17%) used social media monitoring as a way to better understand general public discussions on vaccination, assuming that online discussions are a good proxy for vaccine confidence in a country or region [29,32,34,36-38,45,57,63,65,67,81,95,96,103]. This comes with important limitations due to the lack of representativeness of social media populations. Individuals discussing vaccination on social media tend to come from specific population groups, usually younger or female groups [61]. Furthermore, data extracted from social media platforms are often not representative of the entire online discourse around vaccination on these platforms (see Data Extraction Phase section).

Other objectives included estimating correlations between online activity around vaccination and coverage or outbreak data [40,41,66,104,107,109], describing systems for monitoring vaccination online [28,52,64,97], investigating relations between news media and social media posts [100,108,110], understanding the contribution of bots or trolls to online content about vaccination [31], examining political references to vaccination on social media [35], and detecting anxiety-related adverse events following immunization [89].

#### Addressing Ethical Considerations

Access to social media data is becoming increasingly restricted, as some users set their profiles, conversations, or pages as “private” [111]. Yet, only 15% (13/86) of the studies included in this review were found to have been reviewed and to have received approval from an institutional ethics review board for their study [32,36-38,50,56,67,81,83,85,86,90,93]. An additional 9 studies sent protocols to institutional or ethical review boards and were deemed exempt because they only analyzed public data and social media users were not considered as “human research subjects” [8,31,44,48,60,69,70,75,80]. One study also explained that guidelines from an institutional review board were considered and applied during the study to protect social media users [92].

However, even once ethical approval had been obtained, questions about anonymity, confidentiality, and informed consent remained. For example, one study explained that anonymization of data is extremely difficult to maintain on social media, as content and quotes (whether from private or public data) can easily be traced back to users, revealing their identity [54]. The authors of another study performed on Facebook in Israel explained that although they anonymized their data, informed consent was not required as “subjects would expect to be observed by strangers” when posting messages on the internet [85]. Some studies also discussed the limitations of focusing on public data and the distorted view this creates [28,43,54,61,83,85,88,97,109].

### Data Extraction Phase

#### Selecting Data Extraction Tools and Periods of Monitoring

Studies included in this review were found to use different monitoring tools to extract data from social media platforms. Thirty-five studies used manual browser search tools such as search bars available on Twitter, YouTube, or Facebook [26,60,68-81,83,85,86,89-100,102,104,106,109]. Due to browser and user interface limitations, studies that used manual browser search functions were time-consuming and collected small amounts of data over short periods of time. In one study, the analysis had to be limited to 30 Facebook pages [80], which affected the possibility of capturing data over different periods (ie, possibly missing trends in the number of posts around influenza seasons). Furthermore, owing to the time needed to assess Facebook pages, it was found to be impractical to analyze each complete page in detail. Another limitation comes from browser cookies and personal tracking algorithms imposed by search engines, which can influence manual search results and their listed popularity. Researchers from another study indicated that findings from both the Google and Facebook searches were dependent on the geographic location of the reviewer’s browser settings [89]. Researchers using manual search engines are also restricted to the way search results are presented on different platforms; for example, Pinterest does not list its pins chronologically and does not provide exact time stamps [99]. This made using a more conventional content analysis sampling method (eg, a constructed 2-week time period) virtually impossible in this particular study.

Forty-nine studies used either social media application program interfaces (APIs) (n=24) [29,31-33,35,37-41,43,45,48, 54,56-58,65-67,82,84,87,88,101,103], automated monitoring tools (n=20) [27,36,42,44,46,47,49-53,55,59,61,62,64,105,107, 108,110], or a combination of both (n=5) [8,28,32,34,63] to extract data from social media platforms. The term API refers to a software intermediary that allows two apps to talk to each other [112]. APIs pull and interpret data from servers storing information for Facebook, Twitter, YouTube, Reddit, and many more platforms. It is important to note that APIs do not provide comprehensive access to all social media content, and often only pull random samples of content; for example, Twitter provides access to roughly 1% of public Tweets through its API [113]. Automatic monitoring tools refer to automated web platforms that access social media data via APIs. These automated tools come with user-friendly interfaces, which can be free (with limited access to a random sample of all posts), open source (open to development from other developers), or commercial (where access to a larger percentage of posts is allowed, which can be real-time or archival via a subscription pricing structure). Regardless of the data collection period, studies with the highest number of results and the most robust datasets consistently came from the use of social media APIs or automatic data sampling. The “Yahoo! Answers” API provided the largest sample size from a single platform over a sampling period of 5 years (16 million messages) [103] and Crimson Hexagon was the automated platform that provided the largest mixed sample size, with a mixture of 58,078 Facebook posts and 82,993 tweets over a 7.5-year period [105]. The Yahoo! Answers study found that the API data were difficult to stratify by age, gender, income, education level, or marital status, which may have limited generalizability [103]. Similarly, Smith et al [57] found that using the Twitter API to use social media discussions as a proxy for the population at large is problematic. The difficulty in finding the correct self-assigned demographic of users, and whether they are real users or automated bots, makes the findings less generalizable. Finally, a large number (n=45) of studies using either APIs or automated software focused on Twitter due to the ease of access given by the platform to its data stream compared to other platforms, which may give a skewed perspective of social media attitudes toward vaccines [8,27-59,61-67,105,107,108,110].

Although using a mixture of tools to collect data from social media is possible, only one study used a combination of APIs and manual tools [30].

#### Developing Comprehensive Search Strategies

This review found a diverse range of search strategies developed to extract data from social media platforms. Simple search strategies with one to three keywords were most common. Only 10 studies used more extensive search strategies with Boolean operators to link keywords (eg, AND, OR) or truncations to identify words with different endings (eg, vaccin*) [27,29,42,43,50,51,66,101,105,108]. Although simpler search strategies were perceived as a limitation by some [8,71,73], no data were available on the accuracy of short strategies as opposed to longer and more complex search strategies. Studies that evaluated their search strategies found that the categorization of keywords into “relevant,” “semirelevant,” and “nonrelevant” can increase precision [43], and that keywords should reflect cultural and normative differences [108].

Across all studies, most keywords were related to vaccines (ie, synonyms of the word “vaccine” or brand names of vaccines) and vaccine-preventable diseases. Some studies also searched for adverse events claimed to be linked to vaccination by the public (eg, autism, autoimmune disorders), keywords related to specific controversies (eg, mercury, big pharma, aluminum), or the names of people involved in controversies (eg, Jenny McCarthy, Andrew Wakefield). In addition to keywords, certain studies used hashtags (eg, #vaccine, #cdcwhistleblower, #vaccineswork) [8,30,34,44,48,49,55,60,61,105], questions inputted into search engines (eg, “should I get the HPV [human papillomavirus] vaccine?”) [75,102], or phrases to refer to specific events (eg, “fainting in school children after vaccine”) [89].

Predefined exclusion criteria were also used to screen data and exclude irrelevant or duplicate results. The question of how to deal with data from dubious sources, trolls, or bots was raised, and although researchers in two studies decided to exclude them, two studies specifically analyzed them and acknowledged their impact on the quality and validity of their findings [31,45,57,108].

### Data Analysis Phase

#### Analyzing Metadata, Including Geolocation

The included studies analyzed a range of metadata, from the number of posts to users’ characteristics. Information about the geographical source of social media data was extremely difficult to obtain, as this information was often private, not provided by social media users, or, as one study conducted on Twitter explained, because “accurate location information can be found in only a small proportion of tweets that have coordinates stored in the metadata of the tweet” [40]. This could explain why most of the studies in this review were performed “globally” (n=41).

Despite these challenges, three types of strategies were used to restrict data to certain regions or countries: using keywords in local languages; using location-specific search terms (eg, United Kingdom, Scotland); and directly identifying local or national Facebook groups, pages, or online discussion forums [50,82,86,90,91,93]. Once social media posts were collected, other tools were used to identify and analyze the precise location of data. Some studies manually screened content or collected metadata [39,56,108], whereas others used automated mechanisms and software (Carmen, Geodict, Nominatim, GeoSocial Gauge) to retrieve this information from Twitter [28,29,40,41,54,61,66]. Two studies used dictionaries of terms for geographical entities of countries (GeoNames and the US Office of Management and Budget’s Metropolitan and Micropolitan Statistical Areas) to automatically identify mentions of countries or cities in social media posts or profile pages [29,61]. Some authors also explained that most bots spreading negative content about vaccination online do not report their locations, which could explain why most tweets with geolocation information available were more positive toward vaccination [61].

#### Analyzing Trends, Content, and Sentiments

Once data from social media were extracted from studies, different analyses were performed, ranging from detecting the number of posts available over a period of time to more detailed content analysis to identify the frequency of particular concerns or conspiracies around vaccination [8,27,46-48,53,59,60, 68-71,75-77,79,82,83,90,94,99]. Several studies also performed qualitative thematic analysis [45,56,71,83,85,86,95,99,110], or language and discourse analysis [26,50,52,84,92,93,106]. Four studies compared social media posts to disease incidence or outbreak cases [40,103,105,107].

The most common type of analysis looked at sentiments expressed toward vaccination (70%, 60/86). Sentiments can be understood in a variety of ways, reflected by the range of words identified to designate sentiments toward vaccination across all studies. Most studies used the terms “negative” (n=33), “positive” (n=31), or “neutral” (n=37); however, each study defined these in a slightly different way, which could have influenced study findings and what was perceived by researchers as “negative” or “positive.” Other common sentiments were anti- or provaccine, encouraging or discouraging, ambiguous, or hesitant. Only two studies provided a more comprehensive list of sentiments such as frustration, humor, sarcasm, concern, relief, or minimized risk [53,107]. One study also looked at sentiment as a “yes or no” question: “does this message indicate that someone received or intended to receive a flu vaccine?” [41]. In one study, the World Health Organization determinants of the vaccine hesitancy framework were used to design and test a list of sentiments [90].

Sentiment was determined not only by looking at social media posts or comments but also by coding links, headlines, sources, images, captions, or hashtags [27,35,42,46,60,64,99]. Coding hashtags was sometimes difficult; for example, those using the hashtag #antivaxxers were often denouncing vaccine hesitancy. Similarly, “positive” hashtags such as #provaxxers can be used in a negative context to criticize those who promote vaccination [27]. Coding sarcasm, irony, slang, and hyperboles was also complicated and prone to subjectivity biases [27,32,34,42,44,63,101].

Sentiment analyses were performed manually (40/86, 67%) or using an automated system (19/86, 32%). When data were analyzed manually, studies used multiple coders (between 2 and 4) and assessed interrater reliability scores to ensure accuracy and reliability. Many studies also emphasized the need to provide coders with training and codebooks with precise definitions of codes [8,26,31,32,46,47,53,60,71,73,83,90, 107,109]. Manual sentiment analysis was prone to limitations, particularly because it relied on subjective coding and was labor-intensive, thereby reducing the total number of posts that could be analyzed by a single person [8,43,68,107].

Sentiments were also analyzed using automated systems, with most studies using such systems performed on Twitter (16/19, 84%). Leverage or supervised machine learning was used to code sentiments by training machines to learn how to code different sentiments using a set of manually coded results (ranging between 693 and 8261 posts) [28,34,37-39,41,48, 49,51,57,62,63,66,67,82]. An alternative to manually coding some results to train the machine was to use Amazon Mechanical Turk [41,51,57,66]. Other automated systems that have been used to code sentiments included Latent Dirichlet Allocation, an unsupervised machine-learning algorithm that automatically determines topics in a text [57,101]; Naive Bayes [65]; Lightside [61]; BrightView classifier from Crimson Hexagon [105]; and Topsy [29,44]. Using such programs also came with limitations, including the aptitude of machines to correctly detect sentiments around vaccination, the reliance on manual coding of some part of the data to train the system prone to researchers’ biases and subjectivity, and the need for high computational and technical skills [28,44,101].

#### Assessing the Reach of Social Media

Overall, 49 studies measured potential social media reach and thus estimations of the number of people that see content posted on social media. Reach was determined by the number of followers a user had, as well as the number of engagements with a post (eg, retweeted, shared, saved, liked, and commented upon).

Most studies provided a short descriptive summary of the reach of social media posts, whereas some proposed more detailed or comprehensive analyses. Interactions between different social network communities were studied to understand how information can spread and be shared on social media. Studies found that analyzing retweets was useful to understand the spread of certain sentiments toward vaccination and, in the case of disease outbreaks, to detect how the spread of social media information online can impact vaccination coverage. One particular study investigated how two kinds of communities interacted with each other within conversations about health and its relation to vaccines [62]. From a retweet network of 660,892 tweets published by 269,623 users, the study compared “structural community” with another “opinion group,” and used community detection algorithms and autotagging to measure the interaction, sentiment, and influence that retweets had in conversations between the two communities [62]. Similarly, another study focused on shared concerns about the HPV vaccine and assessed how international followers express similar concerns to those of the groups or individuals they follow [56].

Another study examined communication patterns revealed through retweeting, assessing the impact of various sources of information, contrasting diverse types of authoritative content (eg, health organizations and official news organizations) and grassroots campaign arguments (with the antivaccination community views serving as a prototypical example) [54]. Finally, one study looked at tweeted images, and evaluated predictive factors for determining whether an image was retweeted, including the sentiment of the image and the objects shown in the image [33].

## Discussion

### Principal Findings

Over 80 articles have been published on social media monitoring around vaccination. This growing academic interest, particularly since 2015, acknowledges the role of social media in influencing public confidence in vaccination, and emphasizes the need to better understand the types of information about vaccination circulating on social media and its spread within and between online social networks [114,115]. Social media monitoring still constitutes a relatively new research field, for which tools and approaches continue to evolve. A wide range of methods, varied in style and complexity, have been identified and summarized through this systematic scoping review.

In an effort to summarize media monitoring articles, we developed a three-step model for this review. The first stage, preparation, consists of defining the purpose of social media monitoring while considering any potential ethical issues. The second stage, data extraction, should include the selection of data extraction tools as well as periods of monitoring, and the development of targeted, comprehensive, and precise search strategies. Finally, the third stage, data analysis, could focus on different types of analyses: metadata and geolocation, trends, content, sentiment, or reach. The model was found to be useful in structuring methodologies for social media monitoring, and could be used in the future as a standardized protocol for performing social media monitoring. Further research could be performed to evaluate different components of the model, and propose a more detailed and complex framework for media monitoring.

### Standardization of Social Media Monitoring Methods

Although the large number of articles identified via the scoping review provided sufficient evidence to summarize methods that have been used to monitor social media, almost none of the articles evaluated the precision and accuracy of their monitoring and analysis methodologies. Furthermore, researchers have not drawn on a coherent body of agreed-upon methodologies, and instead created an amalgamation of methodological choices that sets no standards for the right sample size, no recommended time period for different types of analyses per platform, or no recommendations for studying the extremes of positive or negative views (which are not always representative of the general population) [116]. There is also a lack of standardization of which specific API tools or analytical classifiers to use for analyzing social media discourse, interaction, or trends.

There have been recent calls to better standardize social media monitoring methodologies, including practices such as search strategies, so that the quality of the data query is reflected in more accurate and precise data and study findings [117]. However, it may be apt that social media monitoring remains a flexible research design, as the nature and access to social media discourse on vaccination is continuously evolving. The fast-evolving nature of different social media platforms, the crossover of shared data, boundaries to privacy, and public policy surrounding public discourse on vaccination and disease outbreaks may also necessitate a more methodologically diverse approach to keep up with ever-changing developments. Although standardization may not be the best practice for this relatively new research design, there is a need to evaluate the different tools that will be used at each stage of social media monitoring to determine which ones offer the most precise, accurate, and representative results.

### Establishing the Purpose of Social Media Monitoring

Using social media to understand prevailing issues of interest and concern in certain communities can be a useful listening tool for public health institutions, which can then use media monitoring to detect key themes or questions around vaccination circulating in the population. However, many studies included in this review explicitly discussed limitations regarding the lack of population representativeness in investigating social media content. Evidence shows that social media users often represent specific population groups in terms of age, gender, education level, or socioeconomic status [118]. For instance, users discussing vaccination online were found to be younger and female [61]. Another challenge comes from the fact that content being shared by social media users is not always representative of their personal views or feelings, with evidence showing that social media content is often more extreme or impulsive [119]. Due to these issues, social media monitoring is best seen as an alternative to surveys or qualitative interviews in obtaining data about vaccination beliefs and opinions, without assuming representativeness of total populations but rather specific interest groups.

Social media users could also be considered as a configuration of a research population group, and the field of social media monitoring could be seen as an opportunity to understand what information users are exposed to, and how information about vaccination is shared and spread online. In this way, social media monitoring would be used as a new research methodology to study a new type of population. Social media monitoring comes with representativeness challenges of its own, as access to data becomes limited due to inaccessible private content, the challenge of studying all social media platforms at once, or limitations imposed by automated software. However, social media monitoring opens the door to more dynamic research that continuously evolves and responds to a perpetually changing world.

### Important Ethical Considerations

The considerable increase in the number of social media monitoring studies poses questions regarding the safe use of data available online. Even though researchers in previous studies may not have been legally compelled to obtain ethics approval, the lack of guidance on good ethical conduct when using social media information is a cause for concern. Issues of confidentiality and anonymization of data still arise, as some studies included in this review published screenshots of users’ data that included users’ profile names. Another issue relates to data coming from minors, which should be considered more carefully, even when publicly available [120]. Although these concerns should not unduly hinder the development of social media monitoring, they should highlight the need for guidelines to ensure ethical conduct and respect for social media users, and the importance of submitting research proposals to ethics boards.

Recent controversies with regard to the exploitation of users’ data in the Facebook and Cambridge Analytica scandal, and the public outcry of users feeling unnerved being monitored and manipulated, have indeed opened up conversations and legislation around the ethics of handling user data from social media in research [121]. The overall aim of the 2018 EU General Data Protection Regulation (GDPR) is to increase people’s control over their own personal data and “to protect all EU citizens from privacy and data breaches in an increasingly data-driven world” [122]. For companies, organizations, and researchers, this means obtaining consent for using and retaining customers’ personal data, while granting more rights to the “data subject” to be informed and to control how their personal data are used. Such legislation may change the way future researchers must anonymize data, as well as restrict what sections of social media platforms (eg, public vs private) are available for research [123].

### Accessing Data From Different Social Media Platforms: The Twitter Bias

The majority of studies that had the largest datasets, collected over longer time periods, were those with access to social media platforms’ APIs or automated data collection tools. Studies that had smaller samples and used less sophisticated keyword searches were those that relied on manual data collection, and were thus constrained by time, resources, and the limitations of the browser tools used. Although some studies also discussed the time-consuming nature of manual data collection methodologies, the time required to perform searches was not commonly discussed. More research could be performed to evaluate the clear benefits and limitations of manual and automated data extraction tools, including the time required to complete searches. Studies that used the paid version of APIs via automated monitoring software seemed to have a more representative sample, as access to paid data offers access to all historical and current posts. However, there are still issues with the relative opaqueness of the paid access to Twitter, Facebook, or YouTube APIs, which do not advertise the mechanisms behind collection of data, do not inform researchers of what percentage of “all” data they are given, and may thus not provide representative data [124,125]. This also prevents researchers from fully comparing studies over time, as the API sampling algorithm itself will change. It is arguable that such an environment presents risks and opportunities both for data collection strategies in terms of availability and data privacy issues, as well as an evolution in our fundamental understanding of how social media research fits into the rapidly changing public discourse in relation to vaccines. Finally, the financial cost associated with the use of most APIs and automated tools could constitute a barrier to those in lower resource settings [126].

One of the main reasons for the bias toward the use of Twitter in a majority of studies within this review may be because Twitter provides the most openly available API, both for free and with paid access [116]. However, studies using these freely collected tweets only have access to a small (1%) sample of all tweets, creating representativeness challenges [113]. Accessing the free Twitter API also raises issues around periodical collection due to restrictive access to intermittent collection points. This means that any data collection is limited to pockets of time that are not necessarily continuous, truncating the 1% sample into different time periods [127]. This focus on Twitter constitutes an important bias for social media monitoring research, as it fails to capture the real-time evolution of the social media environment, and the flow of users and content from platform to platform [128].

Finally, although subscription-based data analytics companies provide more comprehensive access to other APIs such as YouTube and Instagram, the data provided by these companies can be more skewed toward those of brand marketing (eg, brand strength, brand influencers, and brand trends) [129]. However, there have been growing opportunities in recent years to allow academics to work in partnership with data analytics companies to forge a better understanding of how to look at social media images and text from a social sciences and public policy perspective [130].

### Social Media Analyses: Complexity of Analyzing Sentiments

Although social media data can be analyzed in various ways, ranging from analyzing trends in the number of posts identified to more complex content analyses, most studies focused on sentiment analyses. Identifying sentiments toward vaccination expressed in social media can be useful to detect changes in beliefs and possible drops in confidence. Manual and automated methods of analyzing sentiments both come with their own benefits and limitations: manual coding may be easier and requires less technical skills than automated coding, whereas it is more prone to subjectivity biases and is time-consuming, and therefore does not allow for the analysis of a large number of posts. The complexity of coding discourse, particularly those using sarcasm or irony, is apparent with both automated and manual systems [40]. Researchers should choose analytical methods based on their personal objectives and resources available. Despite the possibility of using a combination of manual and automated coding or selecting automated systems that require less technical skills, more accurate and easy-to-use automated systems should also be developed.

The development of sentiment analysis as a tool in social media monitoring raises other challenges. Most studies identified in this review used simple binary categorizations of sentiments (eg, “negative” vs “positive”). However, discussions around vaccination tend to elicit complex sentiments, closely linked to deeper, more contextual themes of trust, confidence, and risk perception [131]. Categorizing sentiments as either negative or positive therefore fails to recognize nuances that would be crucial for the development of targeted responses to rebuild trust in vaccination. If automated coding systems are to be further developed, they need to take into account the nuances in sentiments around vaccination and move beyond the use of binary variables. More complex sentiment analysis will also improve the quality of the coding of videos, images, and emojis [132-135].

### Considerations for Future Research: Changing Digital Ecosystem

Following the Cambridge Analytica data misuse scandal and an increase in the amount of antivaccine content, Facebook announced a number of API changes aimed at better protecting user information between 2017 and 2019. These restrictions, along with GDPR laws, will pose restrictions on the type of data and research that can be performed on social media platforms and will require researchers to continuously adapt their methodologies [136,137]. Furthermore, platforms such as Pinterest, Facebook, and YouTube are responding to requests from public bodies to alter their content to respond to concerns about the spread of misinformation about vaccines and the presence of antivaccination content on social media [138-140]. These actions from social media platforms may change what users see but also what researchers study. Indeed, it may be that antivaccine groups move away from platforms that no longer monetize or make it easy for them to share information. Those with antivaccination views have not only been found to be using a mixture of websites and social media but also to migrate over to the dark web, where they are able to create and construct content-specific platforms from which their chosen ideologies can be shared [141]. The question of who should decide what content falls under antivaccination sentiment is also important. Social media platforms should work closely with vaccination experts to identify which posts to remove or keep, especially to avoid infringing on the public’s freedom of expression.

### Study Limitations

Some limitations of this systematic scoping review should be acknowledged. Although articles in languages other than English were included for analysis, the search itself only used English keywords, which could have limited the results. Furthermore, the search strategy was comprehensive but did not include certain relevant keywords such as “infodemiology” or “infoveillance,” which should be considered in future research. Data extraction was performed by three researchers, which could have caused inconsistencies even though the same data extraction sheet was used. Finally, social media monitoring constitutes a relatively new research field, which means that many real-life, practical uses of monitoring may have been omitted as they may not have been published in publicly available peer-reviewed journals or reports. It is also important to note that as this is a fast-moving field, a high number of articles have been published since this review was performed, particularly around the COVID-19 pandemic. Methodologies for social media monitoring are expected to continuously and rapidly evolve, and further research should be performed to regularly update this review.

### Conclusion

Social media has changed the communication landscape around vaccination. The increasing use of social media by individuals to find and share information about vaccination, together with the growing volume of negative information about vaccination online, has influenced the way people assess the risks and benefits of vaccination. Social media monitoring studies have been developed with the aim of better understanding the type of information social media users are exposed to, and how this information is spread and shared across the world. This review has identified clear steps to perform social media monitoring that can be organized in three phases: (1) Preparation (defining the purpose of media monitoring, addressing ethical considerations); (2) Data extraction (selecting data extraction tools, developing comprehensive search strategies); and (3) Data analysis (geolocation, trends, content, sentiments, and reach). A wide range of tools for each of these steps have been identified in the literature but have not yet been evaluated. Therefore, to establish social media monitoring as a valuable research design, future research should aim to identify which methods are more robust and precise to extract and analyze data from social media.

## Appendix

Multimedia Appendix 1 Search strategy developed for the EMBASE database.

Multimedia Appendix 2 Summary of included articles.

## Abbreviations

API: application programming interface
ECDC: European Centre for Disease Prevention and Control
GDPR: General Data Protection Regulation
HPV: human papillomavirus
VCP: Vaccine Confidence Project
