# Supplementary material for: Methods for Social Media Monitoring Related to Vaccination: Systematic Scoping Review
Source: JMIR Public Health Surveill. 2021 Feb 8;7(2):e17149. doi: 10.2196/17149 (PMC7899807; doi:10.2196/17149)
Supplement: Multimedia Appendix 1 [file publichealth_v7i2e17149_app1.pdf]

Social media monitoring around vaccination: a systematic scoping review

Multimedia Appendices

Multimedia appendix 1: search strategy developed for the EMBASE database

Search order	List of keywords
1	("health 2.0" or "medicine 2.0" or "web 2.0" or "web 2.0s" or "43 things" or "500px" or "about.me" or "academia.edu" or acfun or advogato or afreecatv or "afreeca tv" or "album2" or android or anobii or aparat or "archive.org" or asianavenue or "asian avenue" or asmallworld or "a small world" or athlinks or "audimated.com" or "baidu tieba" or bayimg or bbm or bebo or bibsonomy or "biip.no" or bilibili or bitchute or blackplanet or "black planet" or "blip.tv" or blog* or "bolt.com" or bookmarksync or "bookmark sync" or "break.com" or busuu or buzznet or cafemom or "care2" or caringbridge or citeulike or "classmates.com" or cloob or "commons.wikimedia.org" or "community manag*" or connotea or couchsurf* or "couch surf*" or cozycot or crunchyroll or cucumbertown or "cyber spac*" or cyberspac* or cyworld or dacast or dailybooth or dailymotion or dailystrength or daum or dayviews or "de.lirio.us" or delirious or "del.icio.us" or delicious or deviantart or diaspora* or digg or diigo or disaboom or "distribution list*" or "dol2day" or doctissimo or dontstayin or douban or doximity or "draugiem.lv" or dreamwidth or dronestagram or "dxy.cn" or "e health" or ehealth or "e-health" or elftown or elixio or ello or engagemedia or "engage media" or "english, baby!" or "eons.com" or etoro or "e-toro" or "experience project" or expotv or "expo TV" or facebook* or faves or fetlife or filmaffinity or "film affinity" or filmow or fledgewing or "fledge wing" or flickr or flixster or "focus.com" or folksonom* or fora or forums or fotki or fotolog* or "fotopic.net" or foursquare or friendica or "friends reunit*" or friendster or fuelmyblog or "funnyordie.com" or funshion or furl or fyuse or "gab.ai" or "gaia online" or gamerdna or "gamer DNA" or "gapyear.com" or "gather.com" or "gays.com" or "gazopa bloom" or "geni.com" or gentlemint or "geograph britain and ireland" or getglue or gfycat or gifboom or girlsaskguys or "girls ask guys" or gnolia or godtube or gogoyoko or goodnesstv or goodreads or goodwizz or googl* or govloop or grindr or "grono.net" or habbo or "hd share" or "hi5" or "hospitality club" or hotlist or "hr.com" or "hub culture" or ibibo or "identi.ca" or imageshack or imessag* or imgur or "imm.io" or "indaba music" or influencer or instagram* or ipad or ipads or ipernity or iphone* or "irc-galleria" or italki or itsmy or jaiku or jalbum or jiepang or "kaixin001" or kakaotalk or "king of glory" or kiwibox or "kodak gallery" or laibhaari or "last.fm" or "late night shots" or "league of legends" or letv or librarything or lifeknot or linkedin* or linkexpats or listography or livejournal or liveleak or livemocha or lockerz or "ma.gnolia" or makeoutclub or mashup* or "mash up*" or mayomo or meetin or meettheboss or meetup or "meet up" or mefeedia or megavideo or mendeley or metacafe or mevio or microblog* or millatfacebook or mixi or "mobileme web gallery" or mocospace or "mouthshut.com" or mubi or mumsnet or "muzu.tv" or "my opera" or myheritage or myspace or "my space" or myvideo or "nasza-klasa.pl" or naver or netlog or "new media" or newgrounds or newsvine or nexopia or "nico douga" or ning or "odnoklassniki" or onedrive or oneworldtv or "online communit*" or "on-line communit*" or

	"open diary" or openfilm or "ora tv" or orkut or outeverywhere or "ovi share" or panoramio or partyflock or patientslikeme or "patients like me" or pearltrees or phanfare or photoblog* or photobucket or "photo sharing" or picasa or pinboard or pingsta or pinterest or pixabay or pixorial or plaxo or playfire or "playlist.com" or plurk or podcast* or poolwo or "qq video" or quechup or quora or qzone or "radar.net" or raptr or ravelry or "rdf site summary" or "really simple syndication" or reddit or rediff or renren or retweet* or "re-tweet*" or "reverbnation.com" or revver or "rich site summary" or "rooster teeth" or "rss feed*" or rumble or rutube or ryze or "sapo videos" or schooltube or sciencestage or "second life" or securetribe or sevenload or sharethemusic or "share the music" or shelfari or shutterfly or simpy or "sina weibo" or sitebar or skoob or skype or skyrock or smartphone* or smugmug or snapchat* or snapfish or "social media" or "social medias" or "social medium" or "social mediums" or "social network*" or socialvibe* or "sonico.com" or soundcloud or "sound cloud" or "spot.im" or "spring.me" or "stage 32" or stickam or streamzoo or streetlife* or "street life*" or "students circle network" or studivz or stumbleupon or talkbiznow or "tape.tv" or "taringa!" or teachstreet or telegram or "tencent qq" or "tencent qzone" or termwiki or "the sphere" or thestudentroom or "the student room" or tinder or tinypic or "travbuddy.com" or travellerspoint or "tribe.net" or trilulilu or "trombi.com" or trooptube or trovebox or tsu or tudou or tuenti or tumblr or "tv uol" or tweet* or twine or twitch or twitter or tylted or unsplash or untappd or uplike or "user generated content" or "vampirefreaks.com" or "vbox7" or veoh or viadeo or viber or viddler or viddsee or videolog* or vidme or vidyard or vimeo or vine or vines or virb or "virtual communit*" or vlog* or vox or wattpad or wayn or "we heart it" or "web 2" or "web page*" or "web site*" or weblog* or webcast* or webmd or webpage* or webshot* or website* or wechat* or weeworld or weibo or wellwer or "wepolls.com" or weread or "wer-kennt-wen" or whatsapp* or wiki* or wistia or wooxie or wordpress or "word press" or "world wide web" or "writeaprisoner.com" or xanga or xing or xmarks or "xt3" or yammer or yelp or yfrog or yookos or youku or youtube* or "you tube*" or zalo or "zing.vn" or "zoo.gr" or zooomr).ti,ab.
2	mobile phone/ or smartphone/ or blogging/ or social media/ or webcast/
3	1 or 2
4	(vaccin* or in*oculat* or immuniz* or immunis* or jab or jabs or shot or shots).ti,ab.
5	vaccination/ or immunization/
6	4 or 5
7	3 and 6
8	limit 7 to yr="2000 -Current"
