# Supplementary material for: Methods for Social Media Monitoring Related to Vaccination: Systematic Scoping Review
Source: JMIR Public Health Surveill. 2021 Feb 8;7(2):e17149. doi: 10.2196/17149 (PMC7899807; doi:10.2196/17149)
Supplement: Multimedia Appendix 2 [file publichealth_v7i2e17149_app2.pdf]

Social media monitoring around vaccination: a systematic scoping review

Multimedia Appendices

Multimedia appendix 2: summary of included articles

REF	Platform	Vaccine	Country	Period monitored	Search strategy	Sentiment analysis
[71]	Facebook	Any	Global, not specified	08/2012 (one point in time)	Manual	Manual (anti, neutral, pro)
[72]	Facebook	Any	Global, not specified	One week in 01/2016	Manual	Manual (anti-vaccination, pro-vaccination, unrelated or unclear)
[73]	Facebook	Any	Italy	07/2009 – 10/2017	Automated (Facebook API[a])	Automated (anger, anxiety, negative, positive)
[74]	Facebook	HPV[b]	United States	06/2016	Manual	Manual (negative, neutral, positive)
[75]	Facebook	Any	United States	01/2015 – 08/2016	Automated (Facebook Python API)	N/A
[76]	Facebook	Polio	Israel	14/08/2013 – 12/11/2013	Manual	N/A
[77]	Facebook	Polio	Israel	28/05/2013 – 31/10/2013	Manual	N/A
[78]	Facebook	Any	Global, not specified	01/01/2010 – 31/05/2017	Automated (Facebook Graph API)	Manual (anti-vaccine, pro-vaccine)
[79]	Facebook	Any (childhood vaccines)	Mix: Australia, United States	14/04/2013 – 14/04/2016	Automated (Facebook API, Social-MediaLab)	N/A
[80]	Facebook	Any	Global, not specified	2007-2016	Manual	Manual (negative, neutral, positive)
[81]	Facebook	Any	Canada	12/12/2013 – 11/01/2014	Manual	Manual (ambiguous, hesitant, negative, positive)
[82]	Forums	H1N1	Spain	N/A	Manual	Manual (sceptical, pro-vaccination)

[84]	Forums	HPV	Romania	2007-2012	Manual (Google Search)	N/A
[85]	Forums	Any (childhood vaccines)	Italy	01/2008 – 06/2014	Manual (Google Search)	N/A
[86]	Forums	Measles	Australia	Three hours	Manual	Manual
[83]	Forums (Babytree.com)	Rotavirus	China	2007-2015	Manual	N/A
[87]	Forums (Iltalehti, KaksPlus)	H1N1	Finland	03/2010 – 05/2010	Manual	Manual
[88]	Forums (www.mothering.com and one unnamed)	Any (childhood vaccines)	Mix: Canada, United States	2004-2012	Manual	N/A
[16]	Forums (www.mumsnet.com)	Measles	United Kingdom	31/08/2000 – 05/03/2003	Manual	Manual (negative, neutral, positive)
[89]	Forums (www.mumsnet.com)	Any	United Kingdom	2011-2017	Manual	N/A
[95]	Mix: Digg, Facebook, Twitter, YouTube	H1N1	Canada	27/10/2009 – 06/04/2010	Manual (Google Search)	N/A
[98]	Mix: Facebook, Forums, Twitter	Measles	Netherlands	15/04/2013 – 11/11/2013	Automated (Twiqs.nl, Howards Home)	Manual (concern, frustration, humour, sarcasm, relief)
[101]	Mix: Facebook, Hyves, LinkedIn, Twitter	Influenza	Netherlands	02/2012 – 04/2012	Automated (Clipit)	N/A
[96]	Mix: Facebook, Twitter	Measles	United States	2009 - 2016	Automated (Crimson Hexagon)	Automated, Brightview classifier (hesitancy, pro-vaccination)
[97]	Mix: Facebook, Twitter	HPV	United States	01/06/2014 – 31/05/2015	Manual	N/A
[100]	Mix: Facebook, Twitter	Measles	Italy	01/01/2010 – 31/12/2015	Manual	Manual (anti-vaccination, neutral, pro-vaccination)
[99]	Mix: Reddit, Twitter	Any	Mix: Canada, United Kingdom, United States	01/02/2015 – 30/09/2016	Automated (Crimson Hexagon's ForSight platform)	N/A

[90]	Pinterest	Any	Global, not specified	19/03/2014-21/03/2014	Manual	Manual (anti-vaccine, neutral, pro-vaccine)
[92]	Reddit	Any	Global, not specified	01/2007 – 09/2014	Automated (Reddit API)	Automated, LDA[c] (affirmative, negative)
[17]	Twitter	Measles	Global, not specified	01/01/2016 – 28/11/2016	Automated (Crimson Hexagon)	Manual (anti-vaccine, pro-vaccine)
[18]	Twitter	Polio and HPV	Global, not specified	10/2012 – 12/2014	Automated (Twitter API+ HealthMap data)	Automated (negative, neutral/unclear, positive)
[19]	Twitter	Pentavalent (DTP-HepB-Hib[d])	Global, not specified	07/2006 – 05/2015	Automated (Twitter API)	Manual (negative, neutral/positive)
[20]	Twitter	Any	Global, not specified	15/04/2014 – 08/11/2014	Automated (Twitter API)	N/A
[21]	Twitter	Any	Global, not specified	04/02/2010 – 10/11/2016 (Study A) & 01/01/2014 – 30/04/2015 (Study B)	Automated (Twitter API, Gnip Inc.)	Manual (anti-vaccine, neutral, pro-vaccine)
[22]	Twitter	Any	Global, not specified	14/07/2014 – 26/09/2017	Automated (Twitter API)	Manual (anti-vaccine, neutral, pro-vaccine)
[23]	Twitter	HPV	United States	07/02/2015 – 13/02/2015	Automated (Twitter API, Python (x,y) and Twython)	Manual (negative, neutral, positive)
[24]	Twitter	Any	Global, not specified	11/11/2014 – 08/08/2016	Automated (Twitter API)	N/A
[25]	Twitter	Any	Italy	01/09/2016 – 31/01/2017	Automated (Twitter API + Java Library: Get Old Tweets)	Automated (in favour of vaccination, neutral, not in favour of vaccination)
[26]	Twitter	Any	United States	01/12/2014 – 01/12/2016	Automated (Twitter API)	Automated (anti-vaccination)
[27]	Twitter	Measles	Global, not specified	01/12/2014 – 30/04/2015	Automated (Discover Text.com)	Automated (negative, neutral, others, positive)
[28]	Twitter	HPV	Global, not specified	15/07/2015 – 17/08/2015	Automated (Twitter API)	Automated (negative, neutral, others, positive)

[29]	Twitter	HPV	Global, not specified	02/11/2015 – 28/03/2016	Automated (Twitter API)	Automated (against, not applicable, pro)
[30]	Twitter	HPV	Global, not specified	01/10/2013 – 01/04/2014	Automated (Twitter API)	Automated (negative)
[31]	Twitter	HPV	United States	01/10/2013 – 30/10/2015	Automated (Twitter API)	N/A
[32]	Twitter	Influenza	Global, not specified	Three influenza vaccination seasons 2013-2016	Automated (Twitter API)	Automated (Does this message indicate that someone received or intended to receive a flu vaccine? (yes, no))
[33]	Twitter	Any	United States	16/04/2015 – 29/05/2015	Automated (Chatter Grabber)	Manual (negative, neutral, positive)
[34]	Twitter	HPV	Netherlands	03/2013 – 04/2013	Automated (Twitter API)	Manual (anti-vaccination, doubt, negative, neutral, no opinion, pro-vaccination, positive)
[35]	Twitter	HPV	Global, not specified	Two weeks in 06/2015	Automated (Topsy)	Automated (Topsy, sentiment score) and manual (negative, neutral, positive)
[36]	Twitter	Influenza	Global, not specified	23/07/2009 – 22/10/2016	Automated (Twitter API)	N/A
[37]	Twitter	Any	Global, not specified	08/01/2012 – 14/01/2012	Automated (NodeXL, Social Media Research Foundation)	Manual (negative, neutral, positive)
[38]	Twitter	HPV	Global, not specified	01/08/2011 – 31/10/2011	Automated (Topsy)	Manual (negative, neutral, positive)
[39]	Twitter	HPV	Global, not specified	01/08/2014 – 31/07/2015	Automated (Twitter API)	Automated (negative, neutral, positive)
[40]	Twitter	HPV	Global, not specified	01/08/2014 – 31/07/2015	Automated (Black and Colleagues	Automated (negative,

					and Microsoft Research)	neutral, positive)
[41]	Twitter	H1N1	United Kingdom	01/04/2009 – 01/05/2010	Automated (Gnip)	N/A
[42]	Twitter	Measles	Global, not specified	01/01/2012 – 30/06/2015	Automated (Twitter Firehose)	Automated (anti, pro)
[43]	Twitter	Any	Global, not specified	01/04/2014 – 20/06/2014	Automated (Vaccine Watch)	N/A
[44]	Twitter	Diphtheria	Spain	01/05/2015 – 15/07/2015	Automated (Topsy)	Manual (negative, neutral, positive)
[45]	Twitter	Measles	Global, not specified	01/02/2015 – 09/03/2015	Automated (Twitter API)	N/A
[46]	Twitter	Any	Global, not specified	11/07/2017 – 17/07/2017	Automated (NodeXL)	N/A
[47]	Twitter	HPV	Mix: Australia, Canada, United Kingdom	01/2014 – 04/2016	Automated (Twitter API)	N/A
[48]	Twitter	Any	United States	08/12/2014 – 02/03/2015	Automated (Twitter API)	Automated, LDA (negative, neutral, positive)
[49]	Twitter	HPV	Global, not specified	10/2013 – 10/2015	Automated (Twitter API)	N/A
[50]	Twitter	Measles	United States	01/12/2014 – 30/04/2015	Automated (Discover Text.com)	N/A
[51]	Twitter	HPV	Global, not specified	01/2016	Manual	Manual (negative, positive)
[52]	Twitter	Any	United States	01/01/2009 – 21/08/2015	Automated (Social Studio's Radian6 API)	Automated, Lightside (anti-vaccine, neutral, pro-vaccine)
[53]	Twitter	Measles	United States	01/02/2015 – 09/03/2015	Automated (Geosocial gauge)	Automated (anti-vaccination, neutral, pro-vaccination)
[54]	Twitter	Any	Italy	01/09/2016 – 30/11/2016	Automated (Twitter API + Java Library: Get Old Tweets)	Automated (negative, positive)

[55]	Twitter	Any	Global, not specified	16/05/2015 – 13/09/2015	Automated (Topsy)	Manual (anti-vaccination, neutral, pro-vaccination)
[56]	Twitter	H1N1	United States	08/2009 – 01/2010	Automated (Twitter API)	Automated, NaiveBayes (negative, neutral, positive)
[57]	Twitter	Measles	United States	2011-2016	Automated (Twitter API)	Automated (anti-vaccine, other, pro-vaccine)
[58]	Twitter	HPV	Global, not specified	01/10/2013 – 31/03/2014	Automated (Twitter API)	Automated (anti-vaccine)
[91]	Weibo	Hepatitis B	China	5/12/2013 – 10/01/2014	Manual	Manual (negative, neutral, positive)
[93]	Yahoo! Answers	HPV	Chile	17/04/2015 – 03/05/2015	Manual	N/A
[94]	Yahoo! Answers	Influenza	Japan	01/04/2004 – 07/04/2009	Automated (Yahoo! Answers API (via Python scripts))	N/A
[59]	YouTube	HPV	Global, not specified	08/02/2008	Manual	Manual (negative, neutral, positive)
[60]	YouTube	HPV	Global, not specified	N/A	Manual	Manual (discouraging, encouraging, neutral)
[61]	YouTube	Any	Global, not specified	04/09/2007 – 17/10/2015	Manual	Manual (discouraging, encouraging, neutral)
[62]	YouTube	HPV	United States	01/11/2010	Manual	Manual (ambiguous, negative, neutral, other, positive)
[63]	YouTube	Any	Global, not specified	15/05-20/05	Manual	Manual (against vaccination, for vaccination)
[64]	YouTube	Any (childhood vaccines)	Italy	06/2014 – 09/2015	Manual	Manual (ambiguous, negative, neutral, positive)
[65]	YouTube	Any (childhood vaccines)	Italy	27/12/2007 – 31/072017	Manual	Manual (negative, neutral, positive)

[66]	YouTube	HPV	Global, not specified	13/11/2006 – 14/04/2014	Manual	Manual (negative, neutral, positive)
[67]	YouTube	Meningococcal B	Global, not specified	19/02/2018 – 21/02/2018	Manual	Manual (ambiguous, negative, neutral, positive)
[68]	YouTube	Any	Global, not specified	20/02/2007	Manual	Manual (ambiguous, negative, positive)
[69]	YouTube	HPV	Global, not specified	26/07/2013	Manual	Manual (negative, positive)
[70]	YouTube	Any	Global, not specified	20/11/2013 - 27/11/2013	Manual	Manual (anti-vaccine, pro-vaccine)

[a] API: Application programme interface
[b] HPV: Human papillomavirus
[c] LDA: Latent dirichlet allocation
[d] DTP-HepB-Hib: diphtheria, tetanus, pertussis, hepatitis B, and haemophilus influenzae type B
